# Effects of Wet Extraction Parameters on Glucomannan Purity and Calcium Oxalate Reduction in Porang (*Amorphophallus oncophyllus*) Tuber Flour

**DOI:** 10.1155/tswj/9982272

**Published:** 2026-04-29

**Authors:** Heny Herawati, Abdullah Bin Arif, Suparlan Suparlan, Feri Kusnandar, Iceu Agustinisari, Sri Widowati, Sintha Suhirman, Misgiyarta Misgiyarta, Muchamad Bachtiar, Hernani Hernani, Atika Hamaisa, Tri Marwati, Fetriyuna Fetriyuna, Fitria Riany Eris, Novitri Hastuti

**Affiliations:** ^1^ Research Center for Process Technology, Research Organization for Energy and Manufacture, National Research and Innovation Agency, South Tangerang, Indonesia, brin.go.id; ^2^ Research Center for Horticulture, Research Organization for Agriculture and Food, National Research and Innovation Agency, Bogor, Indonesia, brin.go.id; ^3^ Research Center for Equipment Manufacturing Technology, Research Organization for Energy and Manufacture, National Research and Innovation Agency, South Tangerang, Indonesia, brin.go.id; ^4^ Food Science and Technology Department, IPB University, Bogor, Indonesia, ipb.ac.id; ^5^ School of Business, IPB University, Bogor, Indonesia, ipb.ac.id; ^6^ Research Center for Food Technology and Processing, Research Organization for Agriculture and Food, National Research and Innovation Agency, Gunung Kidul, Yogyakarta, Indonesia, brin.go.id; ^7^ Department of Food Technology, Faculty of Agro-Industrial Technology, Padjadjaran University, Jatinangor, Indonesia, unpad.ac.id; ^8^ Department of Food Technology, Faculty of Agriculture, Sultan Ageng Tirtayasa University, Serang, Indonesia; ^9^ Research Center for Biomass and Bioproducts, Research Organization for Nanotechnology and Materials, National Research and Innovation Agency, South Tangerang, Indonesia, brin.go.id

**Keywords:** *Amorphophallus onchophyllus,* glucomannan, oxalate, quality, wet extraction

## Abstract

Porang tuber is one of the commodities containing high glucomannan, which increases the body’s immunity and reduces cholesterol, blood sugar, and body weight. However, porang tubers contain calcium oxalate, which hurts the human body. The study aimed to define the effect of soaking ratio, solvent concentration, and processing time on the purification technique to obtain high‐purity flour and determine the best characteristics of porang flour. The porang tubers were extracted using the wet method, which consisted of the immersion ratio of adding sodium metabisulfite (Na_2_S_2_O_5_) and sodium chloride (NaCl) with ethanol solvent at different extraction times. The research resulted in the treatment significantly affecting all observed parameters except fat content in porang glucomannan flour. In addition, the best characteristics were obtained from the treatment of a 1:3 soaking ratio, 96% ethanol concentration, and 120 min of extraction time, which showed a flour whiteness value of 99.38, oxalate of 0.10%, viscosity of 52,758.5 cP, and glucomannan content of 88% on dry basis. Considering the results of all parameters mentioned above, treating a 1:3 soaking ratio, 96% ethanol concentration, and 120 min of extraction time improves the yield and quality of glucomannan flour by decreasing calcium oxalate content by 0.37% from 0.47% to 0.10%. Porang flour with these characteristics is suitable as a thickener or a source of thickening food additives.

## 1. Introduction

Indonesia has abundant natural wealth, which makes it a megabiodiversity country. One of its commodities is porang tubers, which are currently of concern to the country and other countries. Porang tubers (*Amorphophallus oncophyllus*) belong to the Araceae family of tubers [1]. Porang tubers are one of the commodities that contain high levels of glucomannan [2]. Glucomannan is a highly valued financial product. In addition, glucomannan has several benefits, such as being an ingredient in food that improves the body’s immunity [3, 4], lowers cholesterol and blood sugar levels, and reduces weight [5, 6]. Glucomannan also serves as an industrial material [7], including paper‐strengthening agents, glue, edible films, and so on. However, porang tubers contain calcium oxalate (CaC_2_O_4_), which hurts the human body [8].

The CaC_2_O_4_ compound is crystal‐like, with sharp needles embedded in the tissue causing unusual pain. The C_2_O_4_^2-^ with Ca^2+^ minerals in the body produces a nonsoluble and poorly absorbed compound. The intake of CaC_2_O_4_ causes kidney stones in adults [8]. An increase in oxalate food load given to the kidney is suspected of kidney injury [8]. The accumulation of CaC_2_O_4_ can lead to inflammation and kidney disorders.

The porang flour can be extracted mechanically (dry method) and chemically (wet method). The dry method is done by grinding porang tuber slices into glucomannan flour and then purifying it by wind‐sifting. However, porang flour obtained in this way produces low glucomannan purity and is sold as a food ingredient at a low price [10]. Meanwhile, the wet process can be carried out using ethanol to extract porang flour [11]. One of the proven processing technologies that reduce CaC_2_O_4_ levels and increase glucomannan levels is applying NaCl, sodium metabisulfite (Na_2_S_2_O_5_), and ethanol to agricultural products. NaCl solution could decrease the CaC_2_O_4_ levels in purple sweet potatoes [9]. The quality of porang flour can be increased by its glucomannan content by extracting it using ethanol in specific concentrations [11]. However, the extraction method must be compelling because dry materials are used in glucomannan extraction.

While several studies have examined the extraction and purification of glucomannan from porang (*A. oncophyllus*) or konjac, the prevailing literature primarily emphasizes solvent‐based extraction methods, especially those utilizing ethanol or 2‐propanol. These methodologies are predominantly used for desiccated raw materials and are typically refined within a restricted set of processing parameters, including temperature, solvent‐to‐solid ratio, and extraction duration. Prior research has predominantly focused on enhancing glucomannan yield and purity by modifying alcohol concentration and extraction parameters.

However, a substantial gap persists in the literature concerning the concurrent optimization of various critical factors. The synergistic effects of Na_2_S_2_O_5_ and sodium chloride (NaCl) ratios, ethanol concentration, and extraction duration have not been thoroughly examined. This multifactor approach is necessary to gain a deeper understanding of the combination factor between chemical treatments and processing conditions in the purification of glucomannan.

Moreover, a restricted number of studies have investigated the extraction of glucomannan from fresh (wet) porang tubers. This is significant because wet processing offers real benefits for industrial applications, such as eliminating drying stages, reducing energy use, and streamlining processing workflows. Notwithstanding its significance, wet‐based extraction is comparatively underinvestigated relative to traditional dry‐based techniques.

Significantly, the majority of prior research has not explicitly examined CaC_2_O_4_ reduction as a principal response variable. This is a major problem because CaC_2_O_4_ is an antinutritional substance that can be harmful to health by causing irritation and reducing the availability of minerals, which can affect food safety and how well people accept it. Thus, there is a significant need for research that emphasizes reducing CaC_2_O_4_ alongside the purification of glucomannan. So, there still is not a comprehensive study on how to optimize the Na_2_S_2_O_5_:NaCl ratio, ethanol concentration, and extraction time using fresh porang tubers. The study should focus on reducing CaC_2_O_4_ content while maintaining high glucomannan purity.

Improving the quality of porang tuber flour requires efficient reduction in CaC_2_O_4_ levels while concurrently increasing glucomannan content, especially through straightforward, practical processing techniques using fresh (wet) tubers. The use of chemical solvents is a promising method for producing porang flour with reduced CaC_2_O_4_ levels and enhanced glucomannan purity. This study sought to assess the impact of critical processing variables, including the Na_2_S_2_O_5_:NaCl soaking ratio, ethanol concentration, and extraction duration, on the purification of porang flour. The aim was to identify the ideal combination of these elements to yield high‐purity glucomannan flour with diminished CaC_2_O_4_ levels and enhanced physicochemical properties. This study’s findings aim to enhance the safety and quality of porang‐based food products by reducing CaC_2_O_4_, linked to negative health impacts, while improving glucomannan purity. Optimizing the Na_2_S_2_O_5_:NaCl ratio, ethanol concentration, and extraction duration in wet porang tubers is theorized to enhance glucomannan purity while reducing CaC_2_O_4_ levels, thereby elevating the overall quality of porang flour for culinary applications.

## 2. Materials and Method

### 2.1. Plant Material

This study used porang tubers (*A. onchophyllus*) obtained from farmers in Klangon Village, Saradan District, Madiun Regency, East Java Province, Indonesia. The location lies at longitude 111.73° E and latitude 7.51° S. Porang tubers are harvested 7 months after planting and sorted by weight to obtain uniform tubers (2–3 kg).

### 2.2. Extraction of Glucomannan From Porang Tubers

There are several extraction methods in this study, including the immersion ratio of adding Na_2_S_2_O_5_ and NaCl with several treatments (10:10 g, 10:20 g, and 10:30 g) in 1000 mL of water, ethanol solvent with different concentrations (70% and 96%), and extraction time (15, 60, and 120 min). Na_2_S_2_O_5_ technical reagent for food grade and NaCl technical reagent for food grade were obtained from Multi Chemical Indotrading. In extracting porang flour, 350 g of porang tubers were weighed and mixed with ethanol solvent in a blender/extractor. Previously, the tubers were soaked with a specific ratio (Na_2_S_2_O_5_:NaCl) within 1 h. Then, the sample was extracted using a solvent with a particular concentration and extraction time. The extraction treatment on the tubers was carried out with a ratio of the porang tuber sample and the solvent (1:2). After that, the extracted samples were heated in an oven with a temperature of ± 50°C for 24 h. Each treatment purified porang flour under different conditions (Table 1). This study endeavor was structured utilizing a fully randomized design (CRD). The examined parameters included (i) the soaking ratio of Na_2_S_2_O_5_ to NaCl (three levels: 1:1, 1:2, and 1:3); (ii) ethanol concentration (two levels: 70% and 96%); and (iii) extraction duration (three levels: 15, 60, and 120 min), yielding 18 treatment combinations (3 × 2 × 3). The experimental unit was defined as a batch of 350 g of fresh porang tubers undergoing a specific set of treatments, with each unit processed autonomously through the soaking, extraction, and drying phases. Each treatment was replicated twice, yielding a total of 36 experimental units. The replicates were biological, with each replicate using a distinct batch of porang tubers treated under uniform conditions. The porang flour obtained from each treatment was further examined for its features, encompassing glucomannan content, oxalate concentration, physical attributes (whiteness and viscosity), and proximate composition.

**TABLE 1:** Treatment of soaking solution ratio, ethanol concentration, and extraction time of purified porang flour extraction from porang tubers.

Treatments	Soaking (Na_2_S_2_O_5_:NaCl)	Ethanol (%)	Extraction time (minute)
R1C1T1	1:1	70	15
R1C1T2	1:1	70	60
R1C1T3	1:1	70	120
R1C2T1	1:1	96	15
R1C2T2	1:1	96	60
R1C2T3	1:1	96	120
R2C1T1	1:2	70	15
R2C1T2	1:2	70	60
R2C1T3	1:2	70	120
R2C2T1	1:2	96	15
R2C2T2	1:2	96	60
R2C2T3	1:2	96	120
R3C1T1	1:3	70	15
R3C1T2	1:3	70	60
R3C1T3	1:3	70	120
R3C2T1	1:3	96	15
R3C2T2	1:3	96	60
R3C2T3	1:3	96	120

*Note:* R = the immersion ratio of adding Na_2_S_2_O_5_ and NaCl,^R^1 = Na_2_S_2_O_5_ and NaCl (10:10 g), R2 = Na_2_S_2_O_5_ and NaCl (10:20 g), R3 = Na_2_S_2_O_5_ and NaCl (10:30 g), *C* = ethanol concentration, C1 = ethanol 70%, C2 = ethanol 96%, *T* = extraction time, T1 = extraction time 15 min, T2 = extraction time 60 min, and T3 = extraction time 120 min.

### 2.3. Measurement of Glucomannan

One gram of porang flour was added to 50 mL of aquades. The mixture was stirred with constant stirring speed for 2.5 h in an incubator with a temperature of 45°C and a speed of 157 rpm. After that, the mixture was separated from the dregs of porang flour by centrifuging for 20 min at 4000 rpm. The viscous solution and water, separated from the precipitate (dregs), were put into the Erlenmeyer. They added 50 mL of 96% ethanol, poured it slowly, and shook until a precipitate formed, then rinsed again with 50 mL of 96% ethanol. The precipitate was filtered using filter paper heated at 100 °C for 3 h. The precipitate on the filter paper was dried in an oven at 60–80 °C for 24 h until the resulting weight was constant.

Glucomannan content (%) = W2 − W1 sample weight × 100%.

Note: W1 = weight of filter paper; W2 = weight of filter paper + glucomannan (after oven).

The calculation process above is the glucomannan content calculated on a wet basis. The calculation process from a wet basis to a dry basis is done by dividing the wet basis glucomannan content by (100 − the wet basis glucomannan content) and multiplying by 100%.

#### 2.3.1. Measurement of Oxalate Content

Oxalate contents were measured following the method explained by Sarifudin et al. [12]. The sample weighed as much as 1 g, dissolved 50 mL of aquades into the Erlenmeyer, heated at 45 °C for 30 min using ultrasonic, and then centrifuged at 6500 rpm for 10 min, then filtered it. Then, 1 mL of solvent was pipetted using a micropipette, and 1 mL of iron (II) ammonium sulfate, 1 mL of 0.12 M KI solution, a buffer solution with a pH of 5, and 1 mL of KBrO3 were added into a test tube. They were then shaken to homogeneity for 2 min in the acid room. Once homogeneous, the samples were measured with a UV spectrophotometer with *λ* 352 nm.

Oxalate content (%) = A − intercept slope × V (mL) × Fp sample weight.

#### 2.3.2. Measurement of Whiteness

The color quality of porang flour was measured using a chromameter [13]. Parameters measured were L^∗^ (lightness), a^∗^ (+red, −green), and b^∗^ (+yellow, −blue). The Whiteness degree was measured by the formula below. The following calculations for analysis obtained the whiteness degree value:

(1) W=100 – 100−L∗ 2+a∗2+b∗2 0.5.

#### 2.3.3. Measurement of Viscosity

Viscosity was analyzed using the modified method of Shyni et al. [14]. One gram of porang flour slowly dissolved in 100 mL of distilled water in a beaker glass. Then, put the beaker into a container containing hot water at a constant temperature of 80°C over a water bath. The solution was stirred homogeneously for 10 min. Then, it was put into a bottle, cooled at room temperature, and analyzed using a viscometer C and an L4 spindle.

#### 2.3.4. Measurement of Proximate

Measurement of moisture using the drying method in the oven [15]. Analysis of ash content using the furnace method, using the total ash method [14]. Fat content was analyzed using the direct extraction method using soxhlet [15]. Analysis of protein content using the Kjeldahl method [15].

#### 2.3.5. Microstructure Analysis

At this stage, the morphological structure of porang flour in the best extraction treatment was compared with porang flour without extraction (control). The stages of analysis activities are carried out by preparing a tool such as a ZEISS EVO MA 10 SEM (scanning electron microscope). The powder sampel form is coated with carbon tape. The coating process was carried out using a Quorum Type Q150R‐ES Sputter Coater using gold material, sputtering 20 mA, with a sputter time of 60 s. The finished sample is coated with the specimen holder and placed in the testing section of the SEM tool. Next, the ZEISS EVO MA 10 SEM tool carried out the image capture process. The images were taken using a secondary electron (SE) detector and set with a working distance (WD) of 9 to 0 mm and EHT 16.00 kV.

### 2.4. Statistical Analysis

Data were compiled and analyzed using analysis of variance (ANOVA). Further tests were carried out using the Duncan Multiple Range Test (DMRT) at a significance level of 5%. All statistical analysis steps were performed using SAS 9.13 portable software.

## 3. Result and Discussion

### 3.1. Result

#### 3.1.1. Glucomannan and CaC_2_O_4_

In this study, the results showed that there were differences in the average value of glucomannan content in several treatments (Table 2). The Na_2_S_2_O_5_ and NaCl ratio treatment (10:30 g) tended to produce higher glucomannan than the other Na_2_S_2_O_5_ and NaCl comparison treatments. The R3C2T3 treatment produced the highest glucomannan content of 82.08% (Table 2). Ethanol with a concentration of 96% produces a higher concentration of glucomannan than a concentration of 70% when separating glucomannan from impurities. CaC_2_O_4_ is a compound that causes chronic itch in the refining process and will affect the quality of the flour. In this study, the extraction method affected the Ca‐oxalate content in porang flour (Table 2). The Na_2_S_2_O_5_ and NaCl ratio treatment (10:30 g) produced lower Ca‐oxalate than the other Na_2_S_2_O_5_ and NaCl comparison treatments. The oxalate level in treatment R3C2T3 was 0.10%, the lowest compared to other treatments (Table 2).

**TABLE 2:** Means of glucomannan and Ca‐oxalate from various treatments of purified porang flour.

Treatments	Glucomannan (%)	Ca‐oxalate (%)
R1C1T1	63.92 ± 16.76^ab^	0.34 ± 0.15^bcdef^
R1C1T2	71.62 ± 15.89^ab^	0.39 ± 0.15^bcdef^
R1C1T3	73.81 ± 15.21^ab^	0.38 ± 0.15^bcdef^
R1C2T1	65.12 ± 14.21^ab^	0.43 ± 0.15^cdef^
R1C2T2	78.20 ± 16.77^ab^	0.44 ± 0.04^def^
R1C2T3	42.62 ± 19.81^a^	0.47 ± 0.03^f^
R2C1T1	58.86 ± 17.21^ab^	0.32 ± 0.12^bcdef^
R2C1T2	68.72 ± 16.23^ab^	0.30 ± 0.12^bcdef^
R2C1T3	56.17 ± 14.76^ab^	0.25 ± 0.09^abc^
R2C2T1	65.76 ± 13.23^ab^	0.44 ± 0.03^def^
R2C2T2	66.83 ± 16.89^ab^	0.45 ± 0.16^ef^
R2C2T3	72.18 ± 17.66^ab^	0.42 ± 0.11^cdef^
R3C1T1	59.40 ± 15.45^ab^	0.29 ± 0.08^bcde^
R3C1T2	74.07 ± 17.21^ab^	0.22 ± 0.09^ab^
R3C1T3	72.90 ± 13.87^ab^	0.27 ± 0.15^abcd^
R3C2T1	71.60 ± 14.59^ab^	0.29 ± 0.09^bcde^
^R^3^C^2^T^2	78.43 ± 17.71^ab^	0.26 ± 0.14^abc^
^R^3^C^2^T^3	82.08 ± 15.56^b^	0.10 ± 0.03^a^

*Note:* Values are means ± standard deviation, means followed by the different letter in the same column indicate significance by Duncan’s multiple range test (*p* < 0.05). R = the immersion ratio of adding Na_2_S_2_O_5_ and NaCl, R1 = Na_2_S_2_O_5_ and NaCl (10:10 g), R2 = Na_2_S_2_O_5_ and NaCl (10:20 g), R3 = Na_2_S_2_O_5_ and NaCl (10:30 g), *C* = ethanol concentration, C1 = ethanol 70%, C2 = ethanol 96%, *T* = extraction time, T1 = extraction time 15 min, T2 = extraction time 60 min, and T3 = extraction time 120 min.

#### 3.1.2. Physical Characteristics of Purified Porang Flour

The whiteness degree of porang flour is one of the standard requirements for flour quality standards, whose value can be calculated, and the number 100 is expressed as white. The whiteness value of glucomannan flour obtained from various extraction treatments can be seen in Table 3. The R3C2T3 treatment produced the highest whiteness of 99.83% (Table 3). In addition, the extraction method affected the viscosity in porang flour (Table 3). The highest viscosity value obtained in the R3C2T3 treatment was 52,758.5 cP (Table 3).

**TABLE 3:** Means of whiteness and viscosity of purified porang flour.

Treatments	Whiteness (%)	Viscosity (cP)
R1C1T1	92.05 ± 8.72^ab^	25,235.0 ± 6547.0^a^
R1C1T2	91.89 ± 8.49^ab^	34,524.0 ± 7045.5^abc^
R1C1T3	87.91 ± 8.65^ab^	34,579.0 ± 7112.0^abc^
R1C2T1	91.16 ± 8.87^ab^	32,644.5 ± 6970.0^ab^
R1C2T2	93.25 ± 8.67^ab^	39,395.0 ± 7230.5^abcd^
R1C2T3	93.23 ± 8.43^ab^	44,856.0 ± 7543.0^bcd^
R2C1T1	93.84 ± 7.98^ab^	32,281.0 ± 7228.5^ab^
R2C1T2	93.46 ± 7.78^ab^	36,385.0 ± 6980.0^abcd^
R2C1T3	85.21 ± 8.21^ab^	34,700.5 ± 7087.5^abc^
R2C2T1	80.54 ± 8.54^ab^	37,003.0 ± 6965.0^abcd^
R2C2T2	78.24 ± 8.43^ab^	32,952.0 ± 7054.5^ab^
R2C2T3	79.54 ± 7.89^ab^	41,816.0 ± 7211.0^abcd^
R3C1T1	87.89 ± 8.85^ab^	27,877.0 ± 7659.7^a^
R3C1T2	80.76 ± 9.91^ab^	35,086.0 ± 7145.0^abc^
R3C1T3	78.86 ± 13.31^ab^	50,074.0 ± 7025.5^cd^
R3C2T1	77.00 ± 8.87^a^	45,455.0 ± 7976.5^bcd^
^R^3^C^2^T^2	84.90 ± 7.78^ab^	52,055.5 ± 7010.5^d^
^R^3^C^2^T^3	99.38 ± 8.99^b^	52,758.5 ± 7190.5^d^

*Note:* Values are means ± standard deviation, means followed by the different letter in the same column indicate significance by Duncan Multiple Range Test (*p* < 0.05). R = the immersion ratio of adding Na_2_S_2_O_5_ and NaCl, R1 = Na_2_S_2_O_5_ and NaCl (10:10 g), R2 = Na_2_S_2_O_5_ and NaCl (10:20 g), R3 = Na_2_S_2_O_5_ and NaCl (10:30 g), *C* = ethanol concentration, C1 = ethanol 70%, C2 = ethanol 96%, *T* = extraction time, T1 = extraction time 15 min, T2 = extraction time 60 min, and T3 = extraction time 120 min.

#### 3.1.3. Proximate Characteristics

In this study, results of moisture content showed that there was a considerable effect of the extraction method on porang flour, and all treatments showed a moisture content of porang flour < 10% (Table 4). In addition, all treatments showed an ash content of porang flour < 10% (Table 4). The value of fat content did not show a significant difference, with values ranging from 0.21% to 0.64% (Table 4). Then, the extraction method affected the protein content in porang flour, and the average protein content of porang flour ranged from 1.84% to 5.39% (Table 4).

**TABLE 4:** Characteristics of moisture, ash, fat, and protein content of purified porang flour.

Treatments	Moisture (%)	Ash (%)	Fat (%)	Protein (%)
R1C1T1	6.07 ± 1.13^ab^	1.47 ± ^abc^	0.64 ± 0.38^a^	1.84 ± 0.91^a^
R1C1T2	7.43 ± 1.24^ab^	1.20 ± ^a^	0.35 ± 0.21^a^	2.28 ± 0.71^ab^
R1C1T3	7.95 ± 1.26^b^	1.22 ± ^ab^	0.65 ± 0.39^a^	5.39 ± 1.04^c^
R1C2T1	7.63 ± 1.29^b^	1.43 ± ^abc^	0.54 ± 0.29^a^	2.45 ± 0.81^ab^
R1C2T2	7.60 ± 1.31^b^	1.70 ± ^abcd^	0.35 ± 0.17^a^	4.47 ± 1.32^abc^
R1C2T3	7.61 ± 1.23^b^	1.65 ± ^abcd^	0.43 ± 0.18^a^	2.71 ± 1.41^abc^
R2C1T1	7.11 ± 1.17^ab^	1.66 ± ^abcd^	0.31 ± 0.15^a^	3.14 ± 0.97^abc^
R2C1T2	6.66 ± 1.16^ab^	1.28 ± ^ab^	0.30 ± 0.14^a^	2.37 ± 0.87^ab^
R2C1T3	6.11 ± 1.32^ab^	1.59 ± ^abcd^	0.27 ± 0.17^a^	3.51 ± 1.05^abc^
R2C2T1	5.56 ± 1.26^ab^	2.00 ± ^bcde^	0.54 ± 0.21^a^	4.16 ± 1.22^abc^
R2C2T2	4.67 ± 1.23^a^	2.43 ± ^d^	0.61 ± 0.35^a^	3.99 ± 1.37^abc^
R2C2T3	5.86 ± 1.17^ab^	2.29 ± ^de^	0.35 ± 0.15^a^	5.01 ± 1.04^bc^
R3C1T1	7.10 ± 1.22^ab^	1.37 ± ^abc^	0.27 ± 0.18^a^	2.49 ± 0.75^abc^
R3C1T2	7.54 ± 1.12^b^	1.46 ± ^abc^	0.63 ± 0.38^a^	2.10 ± 0.67^ab^
R3C1T3	8.16 ± 1.34^b^	1.37 ± ^abc^	0.35 ± 0.15^a^	2.54 ± 1.11^abc^
R3C2T1	6.92 ± 1.27^ab^	2.12 ± ^cde^	0.26 ± 0.15^a^	2.09 ± 1.21^ab^
R3C2T2	7.53 ± 1.13^b^	2.10 ± ^cde^	0.50 ± 0.26^a^	4.48 ± 1.81^abc^
R3C2T3	6.27 ± 1.33^ab^	1.22 ± ^ab^	0.21 ± 0.16^a^	3.57 ± ^abc^

*Note:* Values are means ± standard deviation, means followed by the different letter in the same column indicate significance by Duncan Multiple Range Test (*p* < 0.05). R = the immersion ratio of adding Na_2_S_2_O_5_ and NaCl, R1 = Na_2_S_2_O_5_ and NaCl (10:10 g), R2 = Na_2_S_2_O_5_ and NaCl (10:20 g), R3 = Na_2_S_2_O_5_ and NaCl (10:30 g), *C* = ethanol concentration, C1 = ethanol 70%, C2 = ethanol 96%, *T* = extraction time, T1 = extraction time 15 min, T2 = extraction time 60 min, and T3 = extraction time 120 min.

#### 3.1.4. Microstructure Characteristics

Based on the analysis of microstructural characteristics using the SEM, quite different results were obtained between the best samples compared to the control. The best‐extracted samples showed that the ethanol extraction resulted in a more crumbly microstructure (Figure 1(a)). In addition, at magnifications of 100–500X, the granules appeared as grains, and needle‐shaped oxalate crystals were not yet visible. Only at a higher magnification of 1000X were the oxalate crystals observed in the control (Figure 1(b)). The oxalate crystals in the control sample still appear sharp and pointed. In contrast, the best extraction treatment (R3C2T3) showed less obvious needle‐shaped crystals.

FIGURE 1The porang flour microstructure using SEM tools (100X, 200X, 500X, 1000X, and 2000X magnification): (a) the best extraction treatment (R3C2T3) and (b) without extraction treatment (control). R3 = the immersion ratio of adding Na_2_S_2_O_5_ and NaCl (10:30 g), C2 = ethanol 96%, and T3 = extraction time 120 min.

**Figure:**
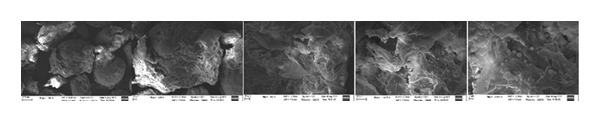
(a)

**Figure:**
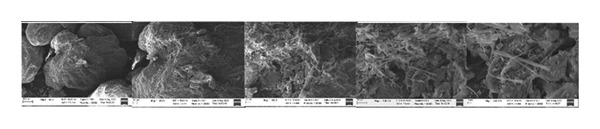
(b)

### 3.2. Discussion

The physical and chemical characterization of porang flour was carried out quantitatively to determine the best soaking ratio, solvent concentration, and duration of extraction time in porang flour high in glucomannan. The characteristics of porang flour were determined after the extraction process (purification) using ethanol. Ethanol is a very polar solvent, volatile, colorless, and nontoxic and can dissolve impurities or unwanted substances. From a physicochemical perspective, the role of ethanol in this system is not only as a general solvent but also as a selective separation medium that modulates solubility based on molecular polarity and intermolecular interactions. In this study, ethanol was used as a washing solution. The purification process involved various treatment ratios (Na_2_S_2_O_5_:NaCl), soaking tubers, solvent concentration, and extraction time. Physical characteristics of porang flour high in glucomannan include analysis of whiteness and viscosity. Chemical characteristics include analysis of glucomannan, CaC_2_O_4_, moisture, ash, fat, and protein content.

In this study, the R3C2T3 treatment produced the highest glucomannan content of 82.08% (Table 2). The best treatment in this study was obtained with a glucomannan content of 82% on a wet basis, which, after being converted to a dry basis, became 88%. Ethanol with a concentration of 96% produced a higher glucomannan content compared to a concentration of 70% when separating glucomannan from its impurities because ethanol dissolves impurities that are not glucomannan. This behavior can be explained by the reduction of the dielectric constant of the solvent system at higher ethanol concentrations, which decreases the solubility of high‐molecular–weight hydrophilic polymers such as glucomannan while enhancing the solubility of lower molecular weight compounds (e.g., sugars, pigments, proteins, and lipids). The 96% ethanol dissolves impurities better than 70% ethanol and leaves glucomannan, which is insoluble in ethanol. Mechanistically, glucomannan tends to form hydrogen‐bonded networks and exhibits limited solubility in ethanol‐rich environments, leading to its selective precipitation, while smaller and less polar molecules remain dissolved and are removed during washing. The 96% ethanol solvent has lower polarity characteristics, so it can dissolve less polar compounds: fat, CaC_2_O_4_, and ash [16]. Therefore, the higher the ethanol concentration, the better the purification process is to produce porang powder with high glucomannan content. Nurlela et al. [2] reported that the higher the ethanol concentration to extract glucomannan from porang tubers, the higher the glucomannan content. In addition, Nurlela et al. [17] reported that the glucomannan content using multigrade ethanol extract with a higher solvent ratio produced a glucomannan content of 90% dryly. The quality requirements of the Republic of China konjac glucomannan are 85% on a dry basis for the first grade and 90% on a dry basis for the higher grade.

In addition, the longer the extraction time, the higher the temperature will increase, with high temperatures increasing the amount of solute dissolved in the solvent, resulting in more extract. In this study, the R3C2T3 treatment produced the highest glucomannan content at an extraction time of 120 min. From a mass transfer standpoint, longer extraction time allows more complete diffusion of soluble impurities from the disrupted tuber matrix into the solvent phase, thereby improving purification efficiency. During the grinding process, the fibers of the peripheral layer are broken down into smaller components; similarly, the starch granules will soften and break into smaller components. This mechanical disruption increases the surface area and enhances solvent penetration into the cellular structure, facilitating the release of entrapped nonglucomannan components. Glucomannan granules are rigid, so they do not separate into small components. This structural rigidity, likely due to semicrystalline regions and strong intermolecular hydrogen bonding, limits their solubilization and helps retain them in the solid phase during ethanol washing. The solvent will carry away small‐sized impurities during separation [18]. Wardhani et al. [19] developed an extraction method using three treatment factors (temperature, extraction time, and 2‐propanol concentration). They recommended an extraction temperature of 75°C for 3 h and a 2‐propanol concentration of 90% as the best treatments. However, the soaking method developed by previous researchers has many shortcomings, such as long extraction time. This extraction technique still requires quite a lot of energy solvents and high temperatures, which are not yet economical and require effort for development on an industrial scale. Guidan et al. [20] reported that glucomannan content decreased with ethanol concentrations above 50% during extraction. In addition, the best extraction time to extract konjac powder with the highest glucomannan content was 60 min. In this study, 96% ethanol treatment and 120 min of extraction time were considered the proper treatment to produce high glucomannan content. This difference suggests that, in wet extraction systems using fresh tubers, the combined effects of solvent selectivity and extended diffusion time may outweigh the limitations reported in dry‐based extraction systems.

In addition to glucomannan content, oxalate content is an essential factor affecting the quality of glucomannan flour. CaC_2_O_4_ is a compound that can cause chronic itching during refining and will affect flour quality. In this study, the oxalate content in the R3C2T3 treatment of 0.10% was the lowest compared to other treatments (Table 2). The reduction of CaC_2_O_4_ can be attributed to several simultaneous mechanisms, including weakening of crystal–matrix interactions, mechanical disintegration, and differential separation during solvent washing. The low oxalate content was caused by soaking with NaCl and purification with high‐concentration ethanol during purification. Increasing the solvent‐starch ratio and filtration time to reach the optimum point during several filtration stages can increase the glucomannan content and reduce the oxalate content in YKF [20, 21]. Wardhani et al. [19] stated that contact time can dissolve impurities that cover particles, thereby increasing the purity of glucomannan. However, increasing the solvent‐powder ratio and extraction time beyond the optimum point during extraction resulted in glucomannan degradation [21, 22]. CaC_2_O_4_ is soluble in water and leached during ethanol extraction [23]. Wardani and Handrianto [24] reported that the optimal results of oxalate reduction in tubers were obtained by soaking in 8% NaCl for 30 min with a soaking rate of ± 5%. In addition, Rofi’ana et al. [9] reported that giving NaCl solution can reduce CaC_2_O_4_ levels. The higher the concentration of NaCl solution, the more significant the reduction in oxalate levels. CaC_2_O_4_ increased when the ethanol concentration and solvent/powder ratio exceeded 50% and 8 mL/g, respectively. In addition, the minimum CaC_2_O_4_ content was obtained at an ethanol concentration of 50% and an extraction time of 60 min at a fixed solvent/powder ratio (8 mL/g) [24]. CaC_2_O_4_ increased when the solvent/powder ratio and extraction time exceeded 8 mL/g and 60 min, respectively. Chua et al. [26] reported that 50% ethanol dissolved impurities in konjac powder. Ethanol concentrations above 50% reduced polysaccharide and impurity deposition [27]. Faridah and Widjanarko [22] reported that extraction time of 3–4 min, stirring speed of 200–400 rpm, and solvent‐powder ratio of 6–8 mL/g, respectively, decreased the concentration of CaC_2_O_4_. Thus, the ratio of Na_2_S_2_O_5_ and NaCl (1:3), ethanol concentration of 96%, and extraction time of 120 min (R3C2T3) produced the highest glucomannan with the lowest oxalate content in this study.

The physical characteristics analyzed in the extracted glucomannan flour were whiteness and viscosity. The degree of whiteness is an important parameter and one of the requirements for the quality of glucomannan flour. Likewise, viscosity is an important parameter because the use of glucomannan is mainly related to its ability as a thickening agent.

The whiteness degree of porang flour is one of the standard requirements for flour quality standards, whose value can be calculated, and the number 100 is expressed as white. The whiteness value of glucomannan flour obtained from various extraction treatments can be seen in Table 3. The whiteness value of glucomannan flour obtained ranged from 77 to 99. The treatment significantly affects the whiteness value of the resulting glucomannan flour. The highest whiteness value was obtained in the treatment using the ratio of Na_2_S_2_O_5_:NaCl (1:3), 96% ethanol concentration, and 96 min of extraction time, equal to 99.38%.

The results of a previous study by Nurlela et al. [17] reported that using ethanol with a higher concentration resulted in a higher degree of whiteness in glucomannan extracted from porang tubers. The highest whiteness degree from this study was 89.91. Previously, Yanuarti et al. [18] reported using a higher ethanol concentration also resulted in a brightness value of 89.48. The increase in brightness and degree of whiteness occurs due to the dissolving of the impurities during extraction.

Sulfites have the ability to inhibit browning reactions, both enzymatic and non‐enzymatic. Several chemicals such as ascorbic acid and citric acid can also inhibit the browning reaction of porang tuber chips [27]. The technique of bleaching porang tuber chips requires ingredients for food‐grade inorganic antibrowning such as Na_2_S_2_O_5_ with a concentration of 0.25% [28] and CaCl_2_ (calcium chloride) with a concentration of 0.1% [26]. Organic antibrowning agents, namely citric acid 0.1%, will be used to prevent discoloration of porang tuber chips during the drying process [29]. Widjanarko et al. [23] obtained the best whiteness degrees (58.91) with the addition of 3% H_2_O_2_. According to Zhao et al. [28], concentrations of organic antibrowning agents such as ascorbic acid were used up to 6%. After doing research and analysis using Na_2_S_2_O_5_ levels exceeding 2%, that is, 7.5%, the results obtained for the whiteness degree values of the use of Na_2_S_2_O_5_ have decreased so that the best results of the whiteness degree value of the acid are at a concentration of 7.5%. PPO enzymes will catalyze the oxidation process of phenol compounds to quinones, which will further polymerize to form brown pigments [30].

The use of Na_2_S_2_O_5_ to prevent browning follows the recommendations by Chua et al. [26]. The use of sulfites is expected to prevent the browning process catalyzed by the phenolase enzyme as well as the consequences of the hydroxyl furfural D‐fix reaction [31]. Based on the results of the research, it shows that the longer the soaking, the whiter degree increases. The N_2_S_2_O_5_ at concentrations of 0%, 2%, 5%, and 7.5% in aquades solution as the antibrowning agent [28]. Using Na_2_S_2_O_5_ can increase the resulting whiteness degree, as indicated by the value of *L* compared to aquades. Using an antibrowning agent, resulted the highest whiteness degree value is obtained using 7.5% Na_2_S_2_O_5_ with increasing maceration time [31].

Viscosity (thickness) is the ability to keep fluid flowing; this analysis is necessary for food/food stabilization [32]. The highest viscosity value obtained in the R3C2T3 treatment was 52,758 cP (Table 3). The higher the soaking rate, solvent concentration, and extraction time, the higher the viscosity. Viscosity is closely related to glucomannan, where viscosity is directly proportional to the amount of the glucomannan produced. The higher glucomannan content, the stickier the solution and thus the higher the viscosity.

The quality standard for konjac flour issued by China has a minimum viscosity characteristic of 28,000 mPas [33]. The best viscosity of porang flour, *A. muelleri* Blume, was produced using extraction with 50% ethanol [34]. While Shimizu and Shimara [35] conducted research on extraction with 30%–80% ethanol to dissolve impurities (starch, fat, protein, oxalate, and ash). An increase in extraction time of 25 min and a solvent/powder ratio of 8 mL/g can increase the viscosity significantly. The viscosity of the glucomannan solution correlates with the purity level of the flour [18]. The impurity components, especially starch, have a characteristic ability to thicken when compared to the ability of glucomannan [10]. The amount of glucomannan in hydrolyzed porang flour may vary depending on the extraction process duration. The glucomannan content in the flour increases with the length of the hydrolysis time [36].

Increasing extraction time and solvent‐to‐powder ratio allows the solvent to interact with the glucomannan particles. As a result, the impurities that coat them are removed, leading to improved glucomannan purity and increased viscosity [37]. However, prolonging the contact time and increasing the solvent/powder ratio beyond the optimal range mentioned above resulted in the degradation of glucomannan. As a result, its purity and viscosity decrease [21]. Thus, comparing Na_2_S_2_O_5_ and NaCl (1:3), the ethanol concentration of 96% and extraction time of 120 min (R3C2T3) produced the highest whiteness and viscosity in this study.

Moisture content is an important parameter for flour products, including glucomannan. Based on Chinese standards, the maximum water content of glucomannan flour is 10% or 3% [38]. WHO standards require a maximum moisture content and ash content of konjac flour to be 15% and 5% [39]. In this study, all treatments showed a moisture content of glucomannan flour < 10% (Table 4). The moisture content correlates with the water molecule contained in flour products and has the potential to become a mold growth medium that can cause damage. High water content can cause the growth of microorganisms and affect other macromolecular components (carbohydrates, proteins, and fats) [40]. Enzymatic reactions can be influenced by other macromolecular components, which result in browning reactions and the emergence of fermentation, so they have the potential to reduce the quality of chips [40]. Soekarto [41] further stated that bound water is divided into primary, secondary, and tertiary bound fractions. Free water evaporates easily and is often found on the surface of foodstuffs.

Besides moisture content, ash content is also an important proximate parameter that determines the quality of glucomannan flour. Ash is an inorganic residue from heating a material at high temperatures > 450°C. Ash content is measured to determine the purity of the material and the number of minerals contained in a material. High ash content in food is caused by more minerals, making it difficult for the digestive system to digest. Chinese standards set the maximum ash for glucomannan flour is 3% [37], and WHO assigned that konjac flour’s maximum ash was 5% [39]. In this study, all treatments showed an ash content of glucomannan flour < 10% (Table 4).

The fat content value will affect the quality and quantity during the storage process of a product. Based on the results presented in Table 4, the value of fat content did not show a significant difference, with values ranging from 0.21% to 0.64%. Therefore, the refining treatment did not significantly affect the fat content of porang flour. Good glucomannan has a low‐fat content [2]. Widjanarko et al. [21] stated that the fat content in porang tuber flour was 2.98%. The refining process will reduce impurity components, including fat, in the resulting porang flour.

Protein is part of the porang root component. In the refining process, it is hoped that the protein will be carried away by the solvent so that the purity of porang flour becomes higher. The average protein content of porang flour ranged from 1.84% to 5.39% (Table 4). Indonesia standards, with number SNI 7938‐2013, set the maximum protein for glucomannan flour ≤ 5%. During the extraction process, the milling process and the presence of ethanol will dissolve the impurity components, including protein, in the solvent so that glucomannan can be easily separated because it is insoluble in ethanol [18].

The purity of glucomannan can be deduced from granule shape, with smoother and more uniform structures signifying more purity [42]. In crude flour, the granules often have a consistent and rather homogeneous oval morphology, while numerous sharp, needle‐like CaC_2_O_4_ crystals remain, which are recognized for inducing discomfort, such as pruritus, in the oral and pharyngeal areas. The extracted materials exhibited notable morphological alterations, including the diminution and absence of oxalate needles during the extraction process. The optimal extraction procedure (R3C2T3) exhibited granules characterized by a reduced quantity and less pronounced needle‐shaped crystals, alongside cleaner, shinier, and more defined surfaces post‐washing [38]. This observation is corroborated by SEM pictures at 2000X magnification, in which the extracted granules exhibited increased transparency. The observed reduction in needle‐like CaC_2_O_4_ crystals in the SEM pictures aligns with the quantitative decrease in Ca‐oxalate content to 0.10%, demonstrating that the extraction method significantly diminished the presence of these irritating chemicals. Thus, the reduced prevalence of sharp oxalate structures is anticipated to diminish irritation and elevate sensory acceptance, thereby augmenting the overall safety and customer preference of the resultant porang flour.

## 4. Conclusion

The study effectively adjusted wet‐extraction parameters for porang flour using a Na_2_S_2_O_5_:NaCl soaking ratio of 1:3, a 96% ethanol concentration, and an extraction duration of 120 min. Under these conditions, the product attained a high glucomannan content (88% on a dry basis), low CaC_2_O_4_ (0.10%), elevated whiteness (99.38%), and viscosity (52,758.5 cP). These attributes align with industrial quality criteria for glucomannan, establishing the flour as a viable thickening agent and a beneficial food ingredient. Future endeavors should focus on process optimization using response surface methods, industrial‐scale validation, and sensory evaluation to improve product applicability and market acceptance.

## Author Contributions

Conceptualization: Heny Herawati, Abdullah Bin Arif, Suparlan Suparlan, Feri Kusnandar, and Iceu Agustinisari. Methodology: Heny Herawati, Abdullah Bin Arif, Suparlan Suparlan, Feri Kusnandar, Iceu Agustinisari, Sri Widowati, Misgiyarta Misgiyarta, Muchamad Bachtiar, Hernani Hernani, and Tri Marwati. Investigation: Heny Herawati, Abdullah Bin Arif, Iceu Agustinisari, Suparlan Suparlan, Misgiyarta Misgiyarta, Atika Hamaisa, Fetriyuna Fetriyuna, Fitria Riany Eris, and Novitri Hastuti. Visualization: Heny Herawati, Abdullah Bin Arif, Iceu Agustinisari, Suparlan Suparlan, Misgiyarta Misgiyarta, Atika Hamaisa, Fetriyuna Fetriyuna, Fitria Riany Eris, and Novitri Hastuti. Data curation: Heny Herawati, Abdullah Bin Arif, Suparlan Suparlan, Misgiyarta Misgiyarta, and Hernani Hernani. Formal analysis: Suparlan Suparlan and Sri Widowati. Writing–original draft: Heny Herawati, Abdullah Bin Arif, Suparlan Suparlan, Feri Kusnandar, Iceu Agustinisari, Sri Widowati, and Atika Hamaisa. Writing–review and editing: Heny Herawati, Abdullah Bin Arif, Suparlan Suparlan, Feri Kusnandar, Iceu Agustinisari, Sri Widowati, Muchamad Bachtiar, Atika Hamaisa, and Tri Marwati.

## Funding

This project was funded by the Indonesia Endowment Fund for Education Agency (LPDP), Ministry of Finance and National Research and Innovation Agency (BRIN) Indonesia for funding this research according to RIIM Grant Nos. 27/III.11/HK/2022 and 17/III.11/HK/2023.

## Conflicts of Interest

The authors declare no conflicts of interest.

## Data Availability

The data used to support the findings of the study are included within the article.
